# Shorter telomere length in COPD cases secondary to biomass-burning smoke exposure

**DOI:** 10.1186/s12931-024-03074-9

**Published:** 2025-01-18

**Authors:** Angélica Domínguez-de-Barros, Gloria Pérez-Rubio, Ingrid Fricke-Galindo, Alejandra Ramírez-Venegas, Malena Gajate-Arenas, Rafael Hernández-Zenteno, Salvador García-Carmona, Robinson Robles-Hernández, María E. Ramírez-Díaz, Filiberto Cruz-Vicente, María L. Martínez-Gómez, Jacob Lorenzo-Morales, Ramcés Falfán-Valencia, Elizabeth Córdoba-Lanús

**Affiliations:** 1 Instituto Universitario de Enfermedades Tropicales y Salud Pública de Canarias (IUETSPC), Universidad de La Laguna, San Cristóbal de La Laguna, Tenerife Spain; 2 Consorcio Centro de Investigación Biomédica en Red (CIBER) de Enfermedades Infecciosas, Instituto de Salud Carlos III, Madrid, Spain; 3 HLA Laboratory, Instituto Nacional de Enfermedades Respiratorias, Ismael Cosío Villegas (INER), Ciudad de México, México; 4 Departamento de Investigación en Tabaquismo y EPOC, Instituto Nacional de Enfermedades Respiratorias Ismael Cosio Villegas (INER), Ciudad de México, México; 5 Coordinación de Vigilancia Epidemiológica, Tlacolula de Matamoros Oaxaca, Servicios de Salud de Oaxaca, Jurisdicción 06 Sierra, Oaxaca, México; 6 Departamento de Medicina Interna, Hospital Civil Aurelio Valdivieso, Servicios de Salud de Oaxaca, Oaxaca, México; 7 Hospital Regional de Alta Especialidad de Oaxaca, Oaxaca, México; 8 Departamento de Obstetricia y Ginecología, Pediatría, Medicina Preventiva y Salud Pública, Toxicología, Medicina Legal y Forense, y Parasitología, Facultad de Ciencias de la Salud, Universidad de La Laguna, San Cristóbal de La Laguna, Tenerife Spain

**Keywords:** Biomass smoke, COPD, Pulmonary function, Telomere length

## Abstract

**Background:**

Chronic obstructive pulmonary disease (COPD) is characterized by progressive airflow obstruction and destruction of lung tissue, primarily attributed to tobacco smoking. However, other factors like biomass-burning smoke (BS) exposure are also implicated. COPD has been described as an accelerated aging disease, and telomere length is a biomarker of aging.

**Methods:**

This study examined telomere length in 189 Mexican individuals, from which 93 developed COPD secondary to BS exposure (BE-COPD); the rest of the participants were exposed to BS but did not develop the disease. Lung function parameters were measured by spirometry, and relative telomere length (rTL) from peripheral blood DNA was determined using multiplex qPCR.

**Results:**

Results showed rTL to inversely correlate with age (R^2^=-0.207, *p* = 0.006) and with the hours-a-day of BS exposure (R^2^=-0.297, *p* < 0.001). Within BE-COPD cases, rTL was associated with daily BS exposure, and BE-COPD individuals exhibited a reduced rTL compared to controls (1.39 ± 0.45 vs. 0.89 ± 0.50; *p* < 0.001). When compared by rTL length in BE-COPD cases, longer telomeres were associated with decreased COPD risk (β = 0.134, 95% CI = 0.053–0.339; *p* < 0.001). However, no significant relationship was found between rTL and clinical or lung function parameters in the BE-COPD group.

**Conclusions:**

This is the first study to document that individuals with COPD secondary to biomass smoke exposure present shorter telomeres than BS-exposed subjects who did not develop the disease.

**Supplementary Information:**

The online version contains supplementary material available at 10.1186/s12931-024-03074-9.

## Background

Chronic obstructive pulmonary disease (COPD) is a pulmonary condition characterized by progressive airflow obstruction and destruction of the lung parenchyma, resulting in impaired oxygen exchange and reduced functional capacity. COPD frequently coexists with other age-related comorbidities such as osteoporosis, cardiovascular disease, lung cancer, diabetes, etc. It is one of the most important causes of morbidity and the third cause of death worldwide, causing 3.23 million deaths in 2019, which affects approximately 10% of the world’s population, with a higher prevalence among adults over 40 years old [1]. 

The leading cause of COPD development is commonly linked to tobacco smoking; however, cases of airflow limitation in non-smokers, relatively similar to COPD, have been identified. Therefore, other risk factors, such as chronic asthma, biomass-burning and environmental smoke exposure, diet, tuberculosis sequelae, and low socioeconomic status, are also associated with airflow limitation [2]. 

Biomass-burning smoke (BS), the product of biofuel combustion in low-income countries where it is used for cooking or heating, impacts over 2.4 billion people globally [3], which could contribute a more significant proportion to the global burden of this disease [4]. In ecological terms, biomass refers to any organic matter. Regarding energy, biomass is any organic matter that can generate it, such as wood, forest residues, or plant materials. Biomass also refers to any organic material used for energy in domestic settings, such as wood burned in wood stoves and wood pellets used in domestic biomass boilers. When burned, biomass releases smoke that contains noxious radicals and particulate matter (PM), which are concerning due to their capacity to damage the distal respiratory airways. Other particles and compounds in environmental pollution can cause chromosome instability, enhancing molecular damage and increasing damage to lung tissue [5–7]. 

Approximately 3 billion people are currently exposed to BS, compared to 1 billion who smoke tobacco, of which 30–40% of COPD cases and nearly 90% of COPD deaths in individuals under 70 years occur in low and middle-income countries. In addition to risk factors such as tobacco smoking, socioeconomic factors also play a determining role in health and longevity. Concerning COPD prevalence, lower household income and education are among the leading contributors [8]. Women and children are the main ones exposed to and affected by biomass combustion while performing domestic tasks, commonly reaching around seven hours a day [5]. Notably, women exposed to household biomass burning are at a two-fold increased risk of developing COPD than women not exposed [9]. The progression of the disease was also reported to depend on socioeconomic factors, as difficulties in accessing healthcare, medical monitoring, or treatment impact life expectancy [10].

Exposure to PM causes cellular stress and inflammation, increasing cytokines, circulating neutrophils, and reactive oxygen species in the respiratory airways, which leads to cellular senescence and DNA damage. COPD is a disease of accelerated aging, and telomere length has been proposed as a biomarker of aging. An accelerated telomere shortening was found in COPD patients who were followed for ten years [11, 12]. Telomeres are specialized structures that cap and protect the ends of chromosomes consisting of tandem “TTAGGG” repeats. Telomeres naturally shorten with cell division, but the rate of telomere shortening can be accelerated by oxidative stress and inflammation responses. Shorter telomeres have been associated with a greater risk of various aging-related diseases like cancer, hypertension, diabetes, and COPD [13]. 

The consequences of general air pollution exposure have been well-determined for the respiratory and cardiovascular systems [14]. However, there is a long way to go in studying the consequences of COPD originating from BS exposure on telomere length and pulmonary functions in low-middle-income countries [15]. 

This study assessed telomere length and lung function parameters in Mexican individuals who developed COPD secondary to exposure to biomass-burning smoke.

## Methods

### Study individuals

#### Inclusion/exclusion criteria

One hundred eighty-nine subjects from rural villages in the Oaxaca highlands and suburban areas in the Tlalpan mayoralty that used biomass for cooking and heating were included in the present study. These individuals were non-smokers and part of the National Program for Equality between Women and Men with COPD timely diagnostic campaign of the Instituto Nacional de Enfermedades Respiratorias Ismael Cosío Villegas (INER) in Mexico City [16]. The subjects corresponded to 93 biomass-burning smoke-exposed COPD cases (BE-COPD) and 96 exposed to BS without COPD as controls (BE-controls). In the second stage, a subset of 82 age- and biomass-exposure-paired individuals (41 BE-COPD vs. 41 BE-controls without COPD) were selected for the genetic study.

Inclusion criteria: We included individuals > 40 years exposed to indoor BS. The cumulative exposure to BS was expressed as hours/year, calculated by multiplying the years of cooking with biomass fuel (wood) by the average daily cooking hours [17]. All patients were new diagnoses identified from the abovementioned campaign, clinically stable, without pharmacological treatment at the sampling time for the respiratory disease or another condition, and diagnosed with COPD if they had a forced expiratory volume in the first second/forced vital capacity (FEV_1_/FVC) ratio < 70%. Lung function clinical variables such as FVC, FEV_1_, the lung’s diffusing capacity for carbon monoxide (DLCO), and the six-minute walking test (6MWT, oxygen desaturation, and 6-MW distance) were measured in all participants following the American Thoracic Society/European Respiratory Society guidelines and regulations [18]. Once subjects were identified as COPD patients and sampling was conducted, they received inhaled respiratory therapy as recommended by the international guidelines [19]. Exclusion criteria include exposure to other than BS or a history of asthma, tuberculosis, pulmonary fibrosis, bronchiectasis, or uncontrolled co-morbidities.

The protocol was approved by the Institutional Committees for Research, Biosecurity, and Ethics in Research of the Instituto Nacional de Enfermedades Respiratorias Ismael Cosío Villegas (INER), with approval codes B11–19, C38-19, and B14-17. All patients read and signed the corresponding informed consent forms before enrolling in the study. This study was performed following the Declaration of Helsinki (2013).

### Blood sampling and DNA isolation

As described [20], peripheral blood samples were collected in tubes with EDTA and centrifuged at 4500 rpm for 5 min. Blood cells were further processed for DNA isolation. According to the supplier’s recommendations, genomic DNA was isolated with the Blood DNA Preparation—Solution Kit (Jena Bioscience, Jena, Germany). The purity and concentration of DNA were evaluated through a NanoDrop™ 2000 spectrophotometer, Thermo Fisher Scientific (Massachusetts, USA).

### Telomere length measurement

Relative telomere length (rTL) was measured using a multiplex qPCR protocol established by Cawthon (2002, 2009) [21, 22]. The qPCR reactions were performed in a final volume of 20 µL using 10 ng of DNA, 0.9 µM of *Telg* and *Telc*, and 0.6 µM of *Albu* and *Albd* primers (Supplementary Table 1). Cycling conditions for telomere length measurement were based on previous publications [12]. DNA from a young control individual with long telomeres was used as a reference sample. All the reactions were performed in triplicates on the iQ Cycler Real-Time PCR Instrument (BioRad). The plates of cases and controls were carried out separately. A standard curve derived from the reference sample serially diluted at concentrations of 1.6^2^, 3.2^1^, 6.4, and 1.28 ng/µl was included in every assay. Intra-plate coefficients of variance (CV) were calculated between the replicates, and samples with CV > 5% were excluded from further analysis. The mean plate efficiency was 90% for the telomere and 95.4% for the albumin assays. Relative telomere length was calculated as a ratio of telomere (T) to albumin (S), as previously described by Cawthon, 2009 using the “ΔΔCp with efficiency correction” calculation method [23]. 

### Statistical analysis

Data are expressed as medium and standard deviation (SD). Normal distribution was assessed for continuous variables through Kolmogorov-Smirnoff and Levene tests. Standardized age (individual age - mean age)/age standard deviation) was used for comparisons between cases and controls of the whole cohort due to the significant difference of this variable within groups. A sub-selection of cases and control individuals paired by age and hours of BS exposure was performed for a second analysis. Logarithmic transformation of relative telomere length (T/S ratio) was performed to reduce variability and assure linearity. Subgroups of BE-COPD cases were defined by the relative telomere length (rTL) tertiles: rTL: short (< 0.57), medium (≥ 0.57, ≤ 1.06), and long telomere length (> 1.06). Comparisons of the clinical characteristics between BE-COPD cases and BE-controls and between subgroups were evaluated using Student’s t-test or the unpaired t-test, Chi-square test, or Fisher’s exact test for continuous and categorical variables as appropriate. The Mann–Whitney U test was used to test the intra-group differences. Pearson’s correlation was used to evaluate the relationship between the variables. Logistic regression analysis was used to determine telomere length and the independent variables associated between cases and controls and within BE-COPD cases. Age, sex, BS exposure (BE), and BMI as confounding variables were used for adjustment.

All statistical analyses were performed in SPSS 25.0 IBM software, where *p*-values < 0.05 were considered significant. Graphs were designed with GraphPad Prism v9.0 (Dotmatics, GraphPad Software, San Diego, California, USA).

## Results

The clinical characteristics of the participants included in the study are represented in Table 1. The subgroup of BE-COPD had the same clinical and lung function characteristics as the whole BE-COPD sample (Supplementary Table 2).

**Table 1:** Clinical characteristics of the BS-exposed COPD cases and BS-non-COPD controls

Variable	BE-COPD (*n* = 93)	(BE-controls) (*n* = 96)	*p*-value
Age (years)	72.61 ± 8.87	59.69 ± 8.50	**< 0.001**
Sex (female %)	91.4	97.9	0.06
BBS exposure/day (hrs)	7.83 ± 4.42	5.42 ± 2.90	**< 0.001**
BBS exposure/year (hrs)	45.88 ± 15.90	46.05 ± 12.52	0.94
BMI (kg/m^2^)	27.05 ± 4.44	28.94 ± 5.60	**0.006**
FEV_1_ (L)	1.03 ± 0.32	2.05 ± 0.40	**< 0.001**
FEV_1_ (% pred)	63.55 ± 18.56	103.97 ± 17.06	**< 0.001**
FVC (L)	1.83 ± 0.53	2.49 ± 0.48	**< 0.001**
FVC (% pred)	85.81 ± 19.23	98.51 ± 14.16	**< 0.001**
FEV_1_/FEV (%)	56.59 ± 9.70	85.87 ± 14.01	**< 0.001**
T/S Ratio	0.89 ± 0.50	1.39 ± 0.45	**< 0.001**

Data are expressed as n (%), mean ± SD. BE-COPD: biomass-burning smoke COPD subjects; BS: Biomass-burning smoke; BMI: body mass index; COPD: chronic obstructive pulmonary disease; Pulmonary function post-bronchodilator: FEV_1_: forced expiratory volume in the first second; FVC: forced vital capacity; % pred: percent predicted; T/S ratio: relative telomere length. The *p*-values < 0.05 were considered significant

Over 85% of the individuals analyzed in the present study were women. Subjects in the BE-COPD group were older and had less BMI than BE-controls. Of the cases with BE-COPD, 2.15% were very severe, 18.28% severe, 59.14% moderate, and 20.43% mild, according to the 2024 GOLD report [18]. In general, BE-COPD individuals showed worse lung function capacity than biomass-exposed BE-non-COPD controls (Table 1). The same results were observed for the subsample of BE-COPD individuals and BE-controls paired by age and biomass exposure (Table 2).

**Table 2:** Clinical characteristics of the subgroup of BE COPD cases and BE non-COPD subjects

Variable	BE-COPD (*n* = 41)	(BE-controls) (*n* = 41)	*p*-value
Age (years)	67.22 ± 7.10	64.83 ± 7.5	0.15
Sex (female %)	85.4	100	**0.03**
BMI (kg/m^2^)	27.65 ± 4.64	28.48 ± 6.82	0.53
BBS exposure/day (hrs)	6.10 ± 2.02	5.27 ± 2.13	0.08
BBS exposure/year (hrs)	43.49 ± 14.90	49.22 ± 13.83	0.08
FEV_1_ (L)	1.11 ± 0.35	1.95 ± 0.36	**< 0.001**
FEV_1_ (% pred)	62.93 ± 17.84	104.57 ± 18.01	**< 0.001**
FVC (L)	1.95 ± 0.62	2.41 ± 0.43	**< 0.001**
FVC (% pred)	86.93 ± 19.42	99.49 ± 14.42	**0.002**
FEV_1_/FEV (%)	55.75 ± 9.56	81.25 ± 16.68	**0.001**
T/S Ratio	0.77 ± 0.44	1.46 ± 0.47	**< 0.001**

Data are expressed as n (%), mean ± SD. This subgroup of individuals was paired by age and hours/day of smoke exposure. BE-COPD: biomass-burning smoke COPD subjects; BBS: Biomass-burning smoke; BMI: body mass index; COPD: chronic obstructive pulmonary disease; Pulmonary function post-bronchodilator: FEV_1_: forced expiratory volume in the first second; FVC: forced vital capacity; % pred: percent predicted; T/S ratio: relative telomere length. The *p*-values < 0.05 were considered significant

**Fig. 1:**
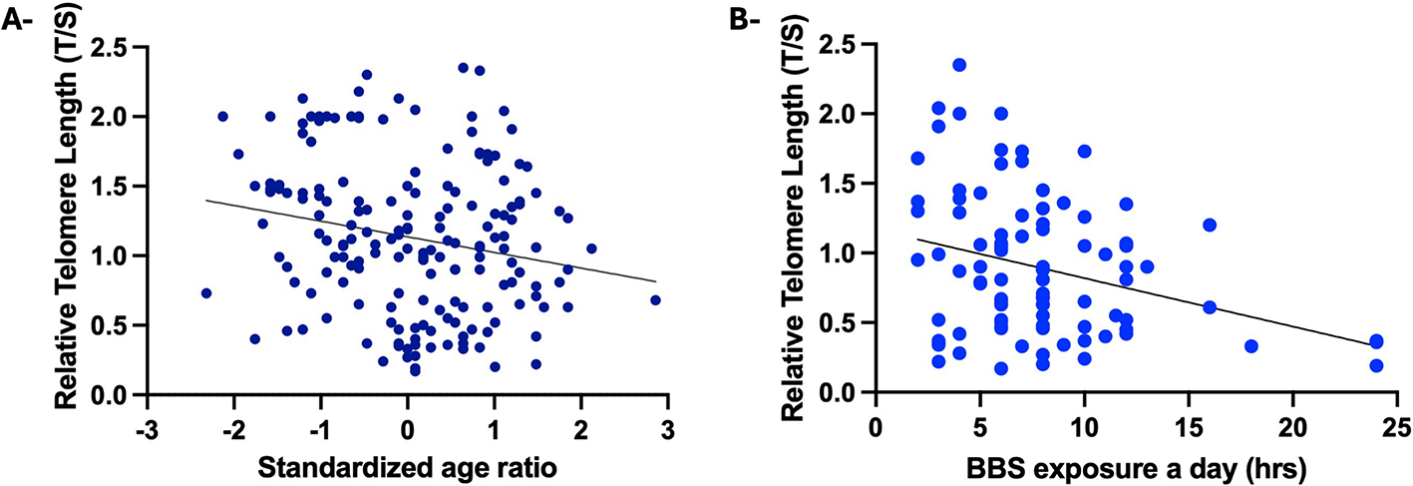
**A**-Correlation between relative telomere length (T/S) and age (standardized age ratio) in the whole cohort of individuals exposed to biomass-burning smoke. **B**-Correlation between relative telomere length (rTL) and the number of hours a day of biomass-burning smoke (BBS) exposure in the BE-COPD group

Telomere length was inversely correlated with age (use of standardized age for comparison between cases and controls) (R^2^=-0.207, *p* = 0.006) (Fig. 1A). TL was also observed to correlate with the hours-a-day BE exposure that the total of individuals exposed to biomass burning smoke (R^2^=-0.297, *p* < 0.001). In the case of individuals with COPD who were daily exposed to biomass smoke, this correlation was increased (R^2^= -0.307, *p* = 0.003) (Fig. 1B).

**Fig. 2:**
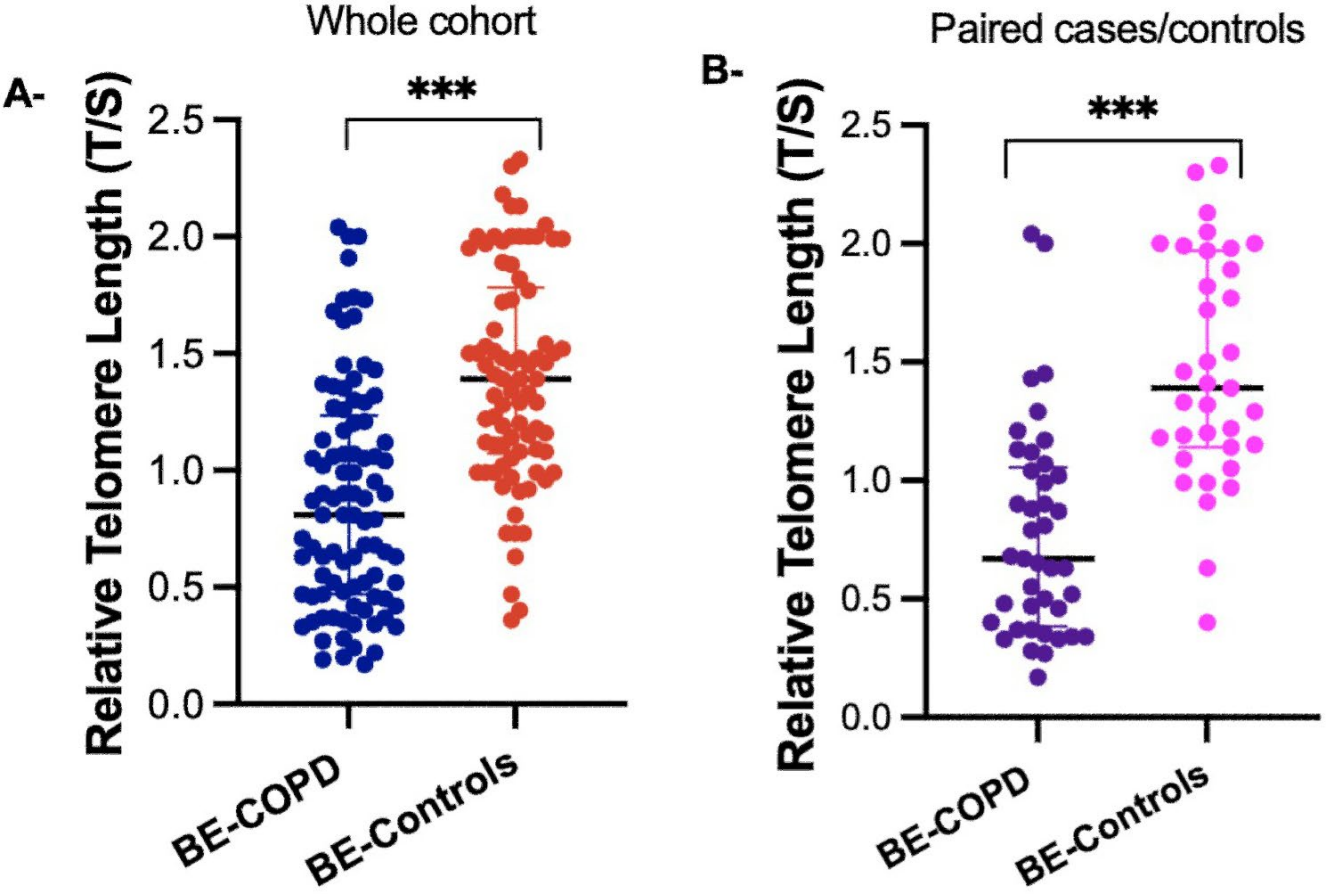
Relative Telomere Length (rTL) was observed in the whole cohort of BE-COPD cases vs. BE-controls **(A).** The same was observed for the subgroup of BE-COPD and BE-controls paired by age and hours/day of biomass-burning smoke exposure **(B).** ***p-value < 0.001

Individuals with COPD secondary to biomass-burning smoke exposure showed reduced rTL compared to BE-controls without the disease (1.39 ± 0.45 vs. 0.89 ± 0.50; *p* < 0.001) (Table 1; Fig. 2). The logistic regression analysis indicated that those individuals with longer telomeres presented a lower risk of developing COPD (β = 0.134, 95% CI = 0.053–0.339; *p* < 0.001, adjusted by age, hours of biomass burn exposure/day and BMI) (Supplementary Table 3). When analyzing the subgroup of individuals paired by age and BE, in those cases with COPD, significantly shorter telomeres were observed in contrast to those individuals BS exposed that did not develop the disease (1.46 ± 0.47 vs. 0.77 ± 0.44; *p* < 0.001) (Table 2; Fig. 2). There was no relationship between telomere length and the clinical and lung function parameters in the BE-COPD cases analyzed in this study.

The BE-COPD group was analyzed according to telomere length. We found that those with shorter telomeres were younger and exposed to more hours/day of BS pollutants. When comparing those BE-COPD cases that presented the shortest telomeres with those with long ones, a slightly reduced airflow capacity, although non-significant, was observed (Table 3and Supplementary Table 4).

**Table 3:** Clinical characteristics of BE-COPD individuals in relation to their telomere length

Variable	Short rTL (*n* = 31)	Medium/Long rTL (*n* = 62)	*p*-value
Age (yrs)	68.35 ± 7.77	74.74 ± 8.67	**< 0.001**
Sex	96.77	88.71	0.19
BMI (kg/m^2^)	27.57 ± 4.02	26.79 ± 4.66	0.22
BBS exposure/day (hrs)	9.27 ± 5.99	7.11 ± 3.20	**0.03**
BBS exposure/year (hrs)	44.58 ± 15.53	46.53 ± 15.79	0.29
FEV_1_ (L)	1.02 ± 0.27	1.05 ± 0.35	0.17
FEV_1_ (% pred)	62.30 ± 17.37	64.16 ± 19.24	0.33
FEV_1_/FVC	57.83 ± 9.33	55.98 ± 9.90	0.19
FVC (L)	1.74 ± 0.30	1.87 ± 0.62	0.08
FVC (% pred)	82.10 ± 15.60	87.67 ± 20.69	0.099

Groups are defined by their relative telomere length (rTL) in tertiles: short and medium + long telomeres. Data are expressed as n (%), mean ± SD. BE-COPD: biomass-burning smoke exposed COPD; BBS: Biomass-burning smoke; BMI: body mass index; COPD: Chronic Obstructive Pulmonary Disease; Pulmonary function post-bronchodilator: FEV_1_: forced expiratory volume in the first second; FVC: forced vital capacity; % pred: percent predicted; T/S ratio: relative telomere length. The *p*-values < 0.05 were considered statistically significant

## Discussion

Air pollution from biomass-burning stoves is one of the world’s most pervasive environmental exposures, affecting the local community, especially women and children. It has been associated with adverse health outcomes, especially in cancer and cardiopulmonary outcomes [13]. This work has deeply characterized the effect of COPD cases exposed to biomass-burning smoke (BS) on telomere length. Our study is the first report that subjects who developed COPD secondary to biomass-burning exposure had shorter telomeres than subjects exposed to BS without any respiratory disease, independently of age and the level of BS exposition.

Air pollution is a significant risk factor for driving respiratory diseases, especially indoor air pollution, which is far more used in low-middle-income countries. It increases the risk of infections and the development of obstructive airway diseases among users and ultimately plays a critical role in inducing oxidative stress and inflammation. Several studies have investigated the relationship between telomere length (TL) and air pollutants. A meta-analysis by Zhao et al. (2018) supports that air pollution is associated with shorter telomere length [14]. An association between reduced telomere length and biomass-burning smoke exposure has been reported. Li et al., 2019 found shorter telomeres in buccal cell samples of women exposed to indoor biomass burn in rural areas of China [13]. Moreover, a study placed in Nepal comparing young children (18–23 months) living in households using biomass as fuel for cooking with children from those where liquefied petroleum gas was used as cooking primarily fuel found significantly shorter telomeres among the first ones [24]. In the same way, a pioneering study by Pavanello et al. in 2010 found shorter rTL in peripheral blood lymphocytes of Polish coke-oven workers exposed to hydrocarbons, suggesting longer and chronic exposure to PAHs as a determinant of shorter TL [7]. Identifying COPD patients due to chronic exposure to chemical products of biomass burning is very important since they are at a potentially increased risk of developing lung cancer.

To the best of our knowledge, no reports explored the association between telomere length and COPD secondary to biomass combustion exposure. Regarding respiratory diseases such as COPD, shorter telomeres were associated with a reduced quality of life and a higher risk of exacerbation and death in patients with moderate-to-severe COPD [25]. Accelerated aging due to telomere shortening may serve as a potential indicator for evaluating COPD progression and life expectancy. This is particularly relevant when considering its impact and convergence with other sociodemographic, lifestyle, and clinical factors that affect patients’ quality of life, especially in low—and middle-income populations [26].

A 10-year longitudinal study showed an association between telomere shortening and worsening of pulmonary gas exchange, pulmonary hyperinflation, and increased mortality risk [11]. In the case of tobacco smokers with COPD, attrition of their telomere length was reported concerning the disease, independent of age and smoking status [2]. In agreement with these previous findings, the current study observed that individuals who developed COPD secondary to biomass combustion exposure had shorter telomeres than BE-control subjects. This work reflects that attrition in telomere length is produced in COPD individuals exposed to BE, the same observed as for those COPD cases secondary to tobacco smoking. Similar biological patterns were observed and described for BE-COPD and tobacco smoking (TS) COPD. Both groups of COPD had a similar risk of exacerbations [27]. In the same way, a similar microbiological and inflammatory profile was observed in BE-COPD as in TS-COPD during exacerbations [28]. 

COPD disease shows heterogeneous and complex phenotypes, progression, and clinical courses. Patients with COPD may have at least four different trajectories in their lung function decline affected/regulated by genetic, environmental, and infectious factors. Little knowledge exists about this in cases of COPD caused by BS exposure [29]. Pial et al., 2020 described the different affections of respiratory function in BE Bangladeshi women, with a significant decline in FEV_1_ and FVC compared to clean gas fuel users [30]. Our results showed worsening lung function parameters in BE-COPD compared to control individuals. Nevertheless, the relation between telomere length and lung function in BE-COPD was not conclusive and needed further studies. We can rule out the potential influence of pharmacological treatment in these findings since the included patients were diagnosed at sampling time and had no previous treatment or medication for their condition.

This study has some limitations. First, BE-COPD cases included in the study were much older than BE-controls; nevertheless, the subsample matched by age and BE allowed us to control for this variable and confirm the current valuable findings. Second, this cross-sectional design may not be adequate for finding significant associations between telomere shortening and lung function parameters. A longitudinal analysis with several time points of measurements and a follow-up cohort characterization is required to properly assess the effect of telomere shortening on clinical and pulmonary function variables in BE-COPD individuals. Third, telomere length was measured in peripheral leukocytes rather than lung tissue. Nevertheless, plenty of evidence shows a correlation between the TL shortening rate of different tissues [31]. 

Lastly, it is important to recognize that measuring a range of molecular markers related to inflammation, oxidative stress, and molecular instability, among others, as reported in other studies about telomere shortening and COPD studies [7, 32, 33], may enhance our understanding for better characterization of the telomere dynamics in the studied population facilitating future monitoring of disease progression. The inclusion of systemic biomarkers in further studies is guaranteed.

## Conclusions

In conclusion, this is the first research to report that COPD cases secondary to biomass-burning smoke exposure present shorter telomeres than subjects exposed to BBS that did not develop the disease. The association found was independent of age and BE level. Further, longitudinal studies with complementary biomarkers measurements and detailed treatment data are necessary to evaluate the impact of telomere shortening on pulmonary function in individuals with BE-COPD.

## Electronic supplementary material

Below is the link to the electronic supplementary material.

Supplementary Material 1

## Data Availability

No datasets were generated or analysed during the current study.
