# Supplementary material for: Shorter telomere length in COPD cases secondary to biomass-burning smoke exposure
Source: Respir Res. 2025 Jan 18;26:23. doi: 10.1186/s12931-024-03074-9 (PMC11742994; doi:10.1186/s12931-024-03074-9)
Supplement: Supplementary file 1 — Supplementary Material 1 [file 12931_2024_3074_MOESM1_ESM.docx]

**Supplementary material**

**Supplementary Table 1.**  Primers used in the multiplex qPCR assay for relative telomere length quantification.

| **Primers** | **Sequence 5´-3´(Cawthon, 2009)** |
| --- | --- |
| **telg** | ACACTAAGGTTTGGGTTTGGGTTTG GGTTTGGGTTAGTGT |
| **telc** | TGTTAGGTATCC CTATCCCTATCCCTATCCCTATCCCTAACA |
| **albu** | CGGCGGCGGGCGGCGCGGGCTGGGCGGaa atgctgcacagaatccttg |
| **albd** | GCCCGGCCCGCCGCG CCCGTCCCGCCGgaaaagcatggtcgcctgtt. |

**Supplementary Table 2.**  Clinical characteristics of biomass-exposed BE-COPD patients included in the study and the selected BE-COPD patients with similar age and biomass-exposure-a-day.

| **Variable** | **BE-COPD (total)** (n=93) | **BE-COPD subgroup** (n=41) | ***p-value*** |
| --- | --- | --- | --- |
| FEV_1_ (L) | 1.038 ± 0.32 | 1.111 ± 0.35 | 0.12 |
| FEV_1_ (% pred) | 63.550 ± 18.57 | 62.927 ± 17.84 | 0.43 |
| FVC (L) | 1.826 ± 0.53 | 1.952 ± 0.63 | 0.12 |
| FVC (% pred) | 85.811 ± 19.23 | 86.92 ± 19.42 | 0.38 |
| FEV_1_/FEV (%) | 56.593 ± 9.70 | 55.751 ± 9.57 | 0.32 |
| BBS exposure/day (hrs) | 7.833 ± 4.42 | 6.098 ± 2.02 | **0.001** |
| BBS exposure/year (hrs) | 45.882 ± 15.98 | 43.488 ± 14.90 | 0.21 |

Data are presented as mean ± SD. BE-COPD: biomass-exposed COPD; BBS: Biomass-burning smoke; BMI: body mass index; COPD: chronic obstructive pulmonary disease; Pulmonary function test post-bronchodilator: FEV_1_: forced expiratory volume in the first second; FVC: forced vital capacity; % pred: percent predicted. The p-values<0.05 were considered statistically significant.

**Supplementary** **Table 3.** Binary logistic regression analysis shows the adjusted effect of differential variables in the studied cohort.

| **Variable** | **OR (Exp β) [95% CI]** | ***p-value*** |
| --- | --- | --- |
| FVC (L) | 0.156 [0.061-0.397] | **<0.001** |
| FVC (% pred) | 0.947 [0.923-0.972] | **<0.001** |
| FEV_1_ (L) | 0.003 [0.000-0.038] | **<0.001** |
| %FEV_1(_% pred) | 0.882 [0.845-0.920] | **<0.001** |
| FEV_1_/FVC | 0.811 [0.754-0.872] | **<0.001** |
| T/S Ratio | 0.134 [0.053-0.339] | **<0.001** |

Data are presented as mean ± SD of clinical variables and the marker T/S for the studied cohort. BE-Controls (n=96) and BE-COPD Cases (n=93) were controlled by age, hours a day of biomass exposure, and BMI. OR: odds ratio; 95% CI: confidence interval at 95%; Pulmonary function post-bronchodilator: FEV_1_: forced expiratory volume in one second; FVC: forced vital capacity; % pred: percent predicted. The p-values<0.05 were considered statistically significant.

**Supplementary Table 4.** Clinical characteristics of BE-COPD individuals grouped by their telomere length.

| **Variable** | **Short rTL ^a^**  (n=31) | **Medium rTL ^b^**  (n=31) | **Long rTL ^c^**  (n=31) | ***p-value*** |
| --- | --- | --- | --- | --- |
| Age (yrs) | 68.35± 7.78 | 75.52 ± 9.51 | 73.97 ± 7.80 | **0.003** ^(a, b)^ **0.03** ^(a, c) (b, c)^ |
| Sex (female %) | 96.77 | 87.09 | 90.32 | 0.38 |
| BMI (kg/m^2^) | 27.57 ± 4.02 | 26.51 ± 4.45 | 27.08 ± 4.45 | 0.65 |
| BBS exposure/day (hrs) | 9.27 ±5.99 | 7.74 ± 3.10 | 6.48 ± 3.23 | 0.50 ^(a,b)^  **0.04** ^(a,c)^  0.76 ^(b,c)^ |
| BBS exposure/year (hrs) | 44.58 ± 16.53 | 47.52 ± 16.08 | 45.55 ± 15.70 | 0.77 |
| FEV_1_ (L) | 1.02 ± 0.27 | 1.01 ± 0.36 | 1.03 ± 0.34 | 0.81 |
| FEV_1_ (%) | 62.30 ± 17.37 | 64.03 ± 17.71 | 64.29 ± 20.90 | 0.90 |
| FEV_1_/FVC | 57.83 ± 9.33 | 55.46 ± 10.66 | 56.50 ± 9.22 | 0.63 |
| FVC (L) | 1.74 ± 0.30 | 1.93 ± 0.75 | 1.82 ± 0.47 | 0.38 |
| FVC (% pred) | 82.10 ± 15.60 | 87.93 ± 20.66 | 87.42 ± 21.05 | 0.44 |

Groups are defined by relative telomere length (rTL) tertiles as short, medium, and long. Data are expressed as n (%), mean ± SD. BE-COPD: biomass-burning smoke exposed COPD; BBS: Biomass-burning smoke; BMI: body mass index; COPD: Chronic Obstructive Pulmonary Disease; Pulmonary function post-bronchodilator: FEV_1_: forced expiratory volume in the first second; FVC: forced vital capacity; % pred: percent predicted; T/S ratio: relative telomere length. ^a,b,c^ The p-values<0.05 were considered statistically significant.
